# Life Satisfaction and Mindfulness in Esports-Involved Gamers in Türkiye: Parallel Mediation of Problematic Online Gaming and Maladaptive Daydreaming

**DOI:** 10.3390/bs16091595

**Published:** 2026-09-08

**Authors:** Seyhan Bekir

**Affiliations:** Department of Psychology, Faculty of Letters, Demiroğlu Science University, 34394 İstanbul, Turkey; seyhanbekir@yahoo.com

**Keywords:** esports, life satisfaction, maladaptive daydreaming, mindfulness, parallel mediation

## Abstract

The present study explores the parallel mediating roles of problematic online gaming and maladaptive daydreaming on the relationship between life satisfaction and mindfulness in esports-involved gamers in Türkiye. This research area has attracted growing scholarly interest due to its potential implications for esports-involved gamers’ mindfulness and life satisfaction. The present study employed a parallel multiple mediation model to investigate the indirect roles of these two mediators in the association between life satisfaction and mindfulness in a sample of esports-involved gamers. A total of 480 esports-involved gamers completed an online survey comprising the Satisfaction with Life Scale, the Mindful Attention Awareness Scale, the Internet Gaming Disorder Scale—Short Form, and the Maladaptive Daydreaming Scale. The data were analyzed using correlation analyses and Hayes’ PROCESS macro (Model 4) with bootstrapping to test the hypothesized mediation model. The findings of the study demonstrated correlations between all these variables. Mediation analyses indicated that both problematic online gaming and maladaptive daydreaming mediated the relationship between life satisfaction and mindfulness, as the direct roles became non-significant once the mediators were included. Interventions designed to enhance mindfulness in esports-involved gamers should therefore address this to decrease maladaptive daydreaming.

## 1. Introduction

Neugarten et al. (1961) were the first to describe life satisfaction as the amount of satisfaction a person has during their whole life, from birth to death. Today, people engage in many behaviors that they like to make themselves happier. These behaviors occur for academic development and obtaining professions (Camcı & Kavuran, 2021; Dost, 2007). Limiting the definition of life satisfaction to work, professional, and academic contexts may be too restrictive. Hong and Giannakopoulos (1994) came up with a new definition of life satisfaction that includes leisure and non-work activities. They also said that the way we act and how we feel in these situations can have a big impact on overall satisfaction. Life satisfaction scales are commonly used across diverse populations, from high school and university students to working professionals (Akcan, 2018; Cavga, 2019). The information on these scales should be changed to suit different ages and stages of life. For instance, when evaluating the life satisfaction of university students, it might be more appropriate to consider free and leisure time outside their academic life rather than time outside work, as stated by Hong and Giannakopoulos (1994). University students, who generally have more free time than high school students, show high rates of online gaming up to 88% (Gökkaya & Deniz, 2014) despite their increased participation in physical and social activities (Ma et al., 2012). The literature uses various terms to describe online gaming’s harmful effects on gamers. These terms include “online gaming addiction,” “internet gaming disorder,” and “problematic online gaming” (Gentile, 2009; Kuss et al., 2018; Škařupová & Blinka, 2016). Although the specific definitions are still being debated, there are differing opinions in the DSM-5 and the ICD-11. The ICD-11 includes gaming disorder, whereas the DSM-5 lists it as a topic requiring further research (APA, 2022; Rumpf et al., 2018).

While online gaming’s adverse effects are not yet classified as an official psychiatric disorder, researchers continue to study this phenomenon (Petry & O’Brien, 2013). Since participants in this study had no formal mental health diagnoses related to addiction or other disorders, and given the lack of a comprehensive medical definition, we will use the term ‘problematic online gaming.’ National and international studies have reported strong negative relationships between problematic online gaming and life satisfaction (Gaetan et al., 2012; Meral, 2017). In terms of predictive associations, however, it is possible to note some differences in opinion; Çelik and Çelik (2022) and Makas and Koç (2025), for instance, analyzed the predictive role of life satisfaction in problematic online gaming and reported that life satisfaction is a significant independent variable. However, many studies have found that problematic online gaming predicts life satisfaction (Pang et al., 2025; Taş, 2025). The hypothesis in this study is that individuals with low life satisfaction are more prone to online gaming due to their escape coping mechanisms or escape motivations (Kardefelt-Winther, 2014; Király et al., 2017); life satisfaction has therefore been used as an independent variable.

However, a research study by Dönmez (2018) revealed that mindfulness can actually help people who have an online gaming addiction lead happier lives. This is different from what other studies have found. The study found that mindfulness mediated the relationship between the variables, with significant correlations among three variables. Other studies also indicate a significant positive relationship between mindfulness and life satisfaction (Güler & Usluca, 2021). Higher mindfulness levels correlate with increased life satisfaction, while lower mindfulness is associated with decreased satisfaction. Research has shown negative correlations between life satisfaction and problematic gaming, and positive correlations between life satisfaction and mindfulness. However, little is known about how adverse events might mediate the relationship between positive life events among esports-involved gamers. Furthermore, the literature suggests that mindfulness significantly predicts online gaming and is particularly effective in reducing problematic online gaming (Li et al., 2017, 2018; Yao et al., 2017).

The dynamics of online games include various emotional responses such as attachment, coping with stress, loneliness in real life, compassion, and avoidance of emotions (Boran & Sürücü, 2022; Oğuz & Şenel, 2022). These emotional responses make it easier for esports-involved gamers to bond with in-game characters, fantasize, and get away from real-life stresses (Snodgrass et al., 2014). When we consider that daydreaming has an important place in childhood and adolescence, it can be said that associating with the characters in the game may lead to distortions in the person’s perception of reality and, therefore, to the mediation of maladaptive daydreaming. The fact that both maladaptive daydreaming and problematic online gaming are associated with similar behavioral problems supports the view that these two conditions may have common characteristics (Pietkiewicz et al., 2018). As a matter of fact, problematic online gaming is significantly associated with negative daydreaming (Öğüt, 2024).

Problematic online gaming and maladaptive daydreaming have similar characteristics. They might both be disturbing activities that have a negative role in an individual’s life satisfaction and mindfulness. In support of this view, maladaptive daydreaming can negatively affect an individual’s academic and professional life and cause a decrease in functionality (Somer, 2002; Somer et al., 2016). Among the disorders associated with maladaptive daydreaming, 76.9% was observed to have attention deficit and hyperactivity disorder, 71.8% anxiety disorder, and 53.9% obsessive–compulsive disorder (Kandeğer et al., 2025; Somer et al., 2016). Individuals with substance addiction were found to have more maladaptive daydreaming than those without (Somer et al., 2020). Also, some studies show it has a high correlation with symptoms of depression and anxiety (Kandeğer et al., 2025). Sharma and Mahapatra (2021) reported that a 16 year old experiencing cyberbullying demonstrated increased problematic online gaming and maladaptive daydreaming behaviors. Nonetheless, more research is needed to define maladaptive daydreaming fully. While no study has directly linked maladaptive daydreaming to life satisfaction, significant associations with anxiety, depression, and stress have been identified (Somer et al., 2020). In addition to this, although all the variables were used in previous studies alongside other psychological factors, they were not examined as parallel mediators. For this reason, this study aims to address a gap in the literature by examining both esports-involved gamers and the variables themselves, thereby providing a more comprehensive understanding.

It is hypothesized that maladaptive daydreaming and problematic online gaming could be critical mediating factors in predicting mindfulness based on life satisfaction. This study examined the following hypotheses:

**H1.**  *A significant relationship exists between life satisfaction, mindfulness, and problematic online gaming.*

**H2.**  *Problematic online gaming and maladaptive daydreaming parallel mediate the relationship between life satisfaction and mindfulness.*

## 2. Materials and Methods

### 2.1. Participants and Data Collection

The survey included semi-professional, hardcore, and competitive players (including those from Faceit and ESEA gaming platforms) who participated in at least one esports tournament within esports organizations. This study focuses on individuals actively involved in esports gaming in Turkey (with or without a prize pool). Participants participated online via Discord, STEAM, and gaming platforms. In this study, all gamers are referred to as esports-involved gamers. The reason it is referred to in this way is that they have participated in esports organizations. An analysis using Fisher’s Z found that with *n* = 480, the power to detect r = 0.15 at alpha = 0.05 (two-tailed) is approximately 0.91. This also demonstrates that the relevant study is highly applicable (Ramseyer, 1979). The descriptive statistics of this study are as follows: 480 individuals completed the research measures on Google Forms with no missing data. We did not offer any money. The mean age was 19.06 years (SD = 2.86). The gender distribution comprised 402 males (83.8%) and 78 females (16.3%). Regarding education, 245 participants (51%) were high school graduates, and 235 (49%) were university graduates. Common method bias was assessed using Harman’s single-factor test in SPSS 27, and the first factor accounted for 17% of the variance, indicating that common method bias was not a serious concern (Podsakoff et al., 2003). Of the 486 initial data points, six cases were removed due to extensive missing responses that disrupted the normal distribution, leaving 480 valid data points for analysis.

#### 2.1.1. Measures Satisfaction with Life Scale (SWLS)

The scale developed by Diener et al. (1985) and then adapted into Turkish by Dağlı and Baysal (2016) was originally designed to measure the life satisfaction levels of individuals. In view of the scale’s reliability studies, its test–retest reliability was measured at r = 0.85, and its item–test correlations ranged between 0.71 and 0.80. An analysis of the CFA fit indices revealed that the indices are construct-valid (chi-square/SD 1.17; RMSEA = 0.0030; CFI = 1.00; NFI = 1.00; NNFI = 1.00; GFI = 0.99; SRMR = 0.019). The study has not been adapted to a clinical sample. The measured reliability value in the current study and scale was found to be 0.86.

#### 2.1.2. Internet Gaming Disorder Scale—Short Form (IGDS9-SF)

The scale, which was developed by Pontes and Griffiths (2015) and adapted into Turkish by Arıcak et al. (2018), was developed to determine the problematic online gaming levels of individuals. Because of the scale’s reliability studies, its test–retest reliability was calculated to be r = 0.78, and its item–test correlations ranged between 0.39 and 0.71. CFA fit indices were examined to determine validity (chi-square/SD 4.79; RMSEA = 0.09; CFI = 0.90; TLI = 0.87). The study has not been adapted to a clinical sample. The measured reliability value in the current study and scale was found to be 0.73.

#### 2.1.3. Mindful Attention Awareness Scale (MAAS)

The Mindful Attention Awareness Scale (MAAS) was developed by Brown and Ryan (2003) and later adapted into Turkish by Özyeşil et al. (2011). The fit indices from the Confirmatory Factor Analysis are as follows: RMSEA = 0.06, CMIN/df = 1.09, GFI = 0.93, CFI = 0.90, and AGFI = 0.91. The reliability of the scale was indicated by a Cronbach’s Alpha coefficient of 0.78 in previous studies, while the current study found a Cronbach’s Alpha of 0.91, demonstrating strong internal consistency. The study has not been adapted to a clinical sample. The measured reliability value in the current study and scale was found to be 0.89.

#### 2.1.4. Maladaptive Daydreaming Scale (MDS-16)

Developed by Somer et al. (2016), the MDS-16 was the first scale developed to measure maladaptive daydreaming. The Cronbach’s Alpha reliability coefficient for the original form of the scale was determined to be 0.95. In analyses conducted by Metin et al. (2022) on the form adapted to Turkish culture, the Cronbach’s Alpha reliability coefficient was found to be 0.89. The study has not been adapted to a clinical sample. According to the scores obtained from the scale, individuals scoring 50 or higher are considered to have maladaptive daydreaming. The measured reliability value in the current study and scale was found to be 0.75.

### 2.2. Data and Statistical Analysis

Prior to conducting a statistical analysis, it is imperative to examine the data for any potential outliers or anomalies, as non-normally distributed data can result in erroneous conclusions (Tabachnick et al., 2007). We also examined kurtosis, skewness, VIF, and CI values. Following this, Pearson correlation was used for correlation analyses, and mediation was tested using IBM SPSS Process Macro v5.0.

A parallel mediation model was tested to determine the mediating roles of problematic online gaming and maladaptive daydreaming. Using the SPSS PROCESS (Model 4) to analyze the data. No multicollinearity issues were detected, and the data were normally distributed. Skewness and kurtosis values were considered for the normal distribution. The findings, including Pearson correlation coefficients among the variables and mediation analysis results, are summarized in Table 1.

Jondeau and Rockinger’s (2003) study observed that in the theoretical framework, normality is defined as the premise that the skewness and kurtosis values should not be greater than 3. In this study, the skewness and kurtosis values range from 0.92 to −0.69. It can therefore be concluded that the data are normally distributed. A further analysis was conducted to assess the Variance Inflation Factor (VIF) and Condition Index (CI) values for the presence of multicollinearity. In this article’s findings, it was discovered that it said that it was acceptable (Montgomery et al., 2021). As a result, no such multicollinearity was identified between the variables in this study. Therefore, no problems were encountered in reporting with the HAYES PROCESS (Hayes, 2018). The findings revealed a significant and negative correlation between mindfulness and problematic online gaming (r = −0.24, *p* < 0.01) and maladaptive daydreaming (r = −0.23, *p* < 0.01). This finding signifies that higher levels of mindfulness are associated with reduced scores in both problematic gaming and maladaptive daydreaming. Furthermore, a positive correlation has been found between mindfulness and life satisfaction (r = 0.12, *p* < 0.01), suggesting that individuals who score higher in mindfulness also tend to report higher overall life satisfaction. In the context of these correlations, the correlation between mindfulness and problematic online gaming is observed to be the highest (r = −0.24), with a close correlation observed between maladaptive daydreaming and problematic online gaming (r = 0.50).

## 3. Results

### Parallel Mediation Role of Maladaptive Daydreaming and Problematic Online Gaming

**Figure 1:**
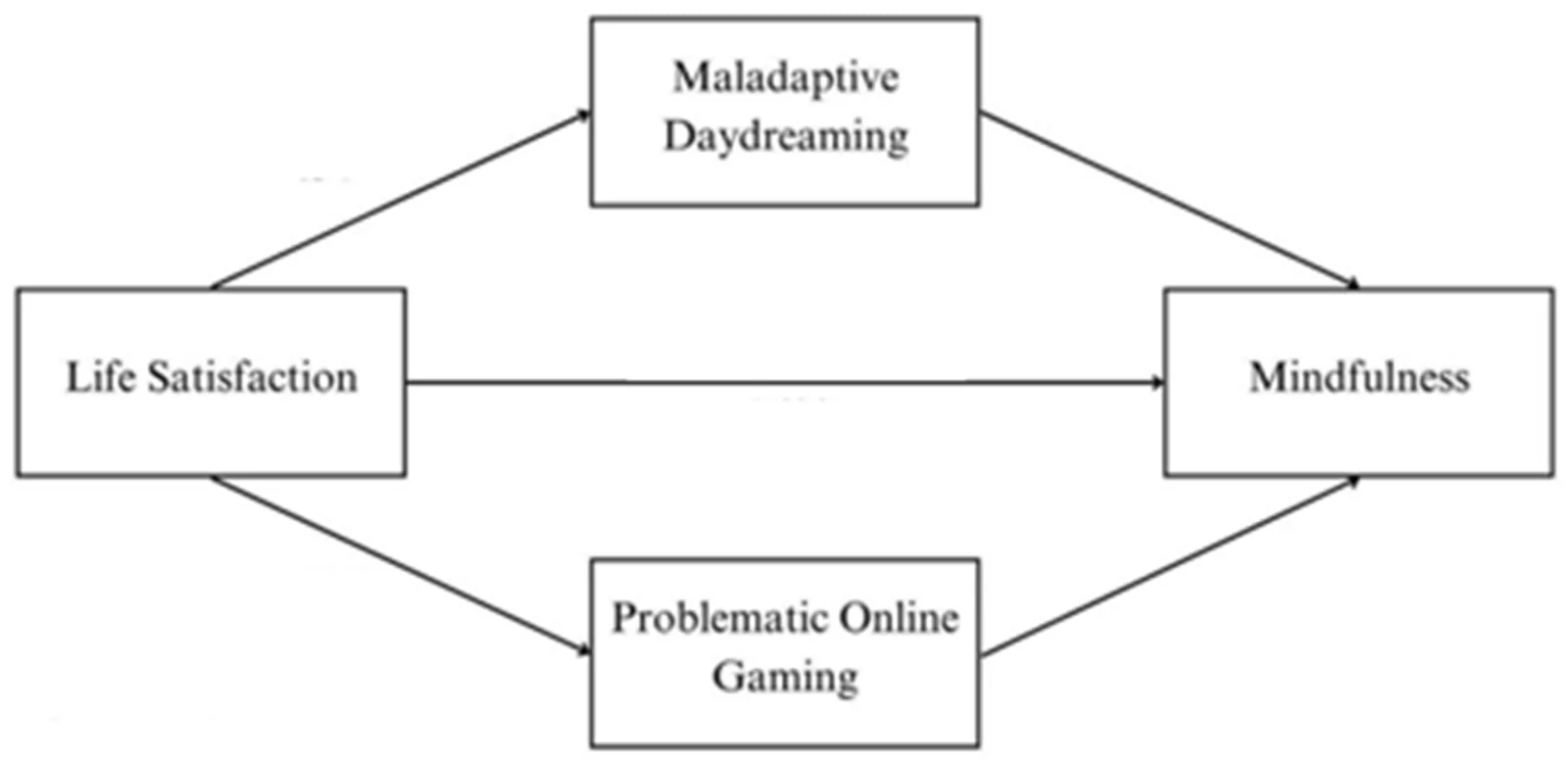
Hypothesized parallel multiple mediation models of the roles of life satisfaction on mindfulness through maladaptive daydreaming and problematic online gaming.

The results regarding the parallel mediating role of problematic online gaming and maladaptive daydreaming are shown in Table 2 and Figure 1. They present the results of Hypothesis 2, illustrating the parallel mediational model coefficients derived from the Hayes PROCESS macro.

**Figure 2:**
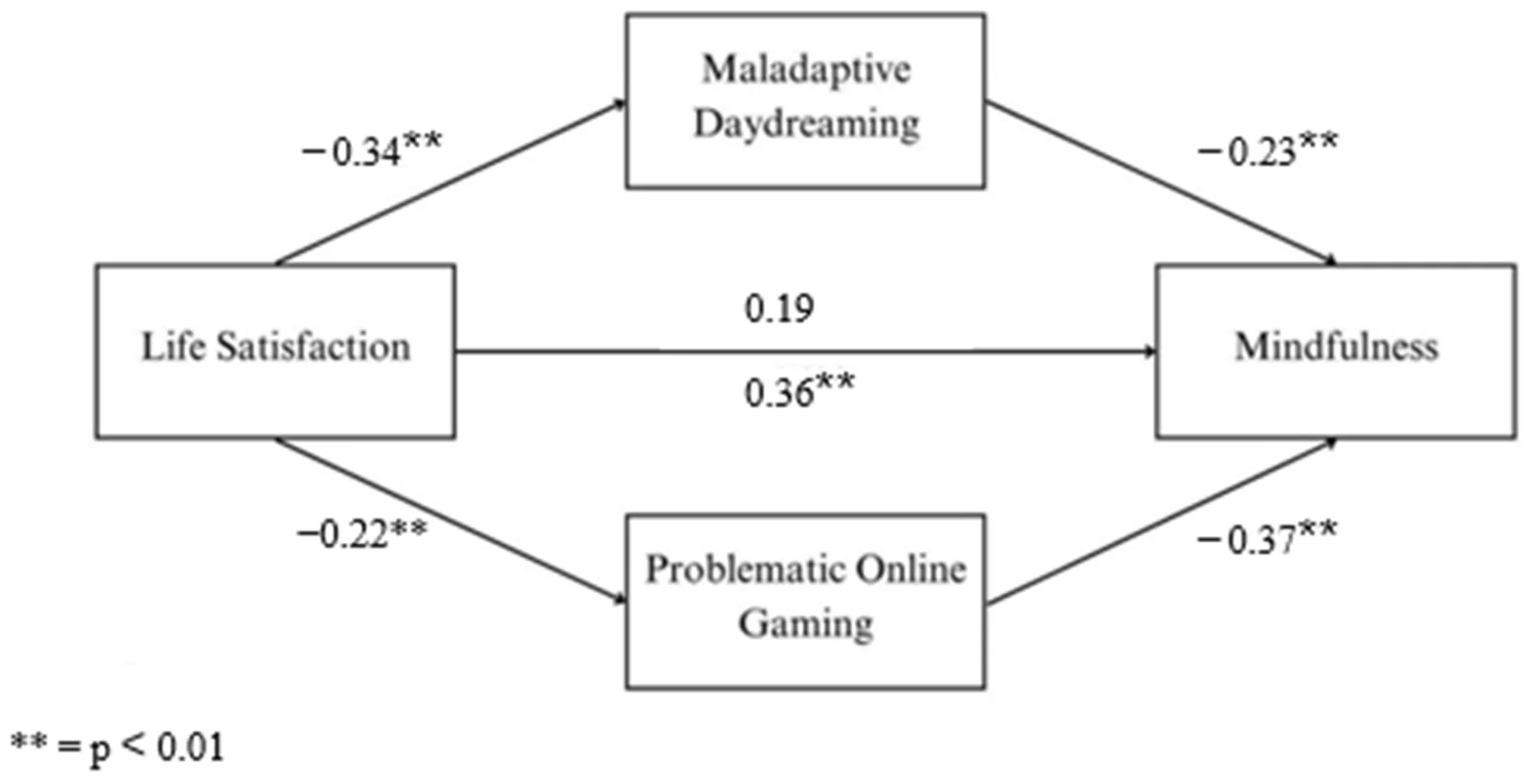
Parallel multiple mediation model showing the indirect effects of life satisfaction on mindfulness through maladaptive daydreaming and problematic online gaming.

A statistical diagram of the parallel multiple mediator model for mindfulness is shown in Figure 2, Table 2 and Table 3. The hypothesized parallel mediator model shown in Figure 1 was tested using Hayes’ PROCESS macro (Model 4) with 5000 bootstrap samples (Bozkurt, 2023; Hayes, 2018). Our initial analysis examined the direct paths of life satisfaction on mindfulness, life satisfaction on maladaptive daydreaming and problematic online gaming (path a^1^b^1^a^2^b^2^, Figure 2), and lastly, maladaptive daydreaming and problematic online gaming on mindfulness. Supporting the direct paths on mindfulness from all variables, life satisfaction (LS) was found to be significantly negatively associated with both maladaptive daydreaming (MD; B = −0.34, *p* < 0.001) and problematic online gaming (POG; B = −0.22, *p* < 0.001). Although the total paths of life satisfaction on mindfulness were significant (B = 0.3617, *p* = 0.001), these direct paths became non-significant when the mediators were included in the model (B = 0.19, *p* = 0.14). In line with the results of both, problematic online gaming (B = −0.37, *p* = 0.001) and maladaptive daydreaming (B = −0.23, *p* = 0.01) were significant negative predictors of mindfulness, suggesting that both function as mediators in the relationship between life satisfaction and mindfulness.

A bootstrapping process of the partial model shown in the indirect roles of life satisfaction on mindfulness through maladaptive daydreaming and problematic online gaming (path a^1^, b^1^; path a^2^b^2^; Figure 2). The findings reported in Table 3 and Figure 2 show that the indirect roles were statistically significant (path a^1^b^1^= 0.0809; boot 95% LL-UL [0.0175, 0.1646]; path a^2^b^2^ = 0.0843; boot 95% LLUL [0.0242, 0.1709]). The total indirect roles were also found to be significant (a^1^b^1^ + a^2^b^2^ = 0.1652; boot 95% LL-UL [0.0803, 0.2686]). Furthermore, all single paths of the mediation model were in the expected direction (see Table 2 and Table 3). However, when both mediators were included in the model, the direct path of life satisfaction on mindfulness (path c′) was no longer statistically significant (B = 0.1965, 95% CI [−0.0676, 0.4605], *p* = 0.1444), indicating mediation.

In summary, the findings indicate that the results of the study demonstrated a significant decrease in life satisfaction in individuals who engaged in maladaptive daydreaming and problematic online gaming. It has been demonstrated that higher levels of maladaptive daydreaming and problematic online gaming are associated with lower levels of mindfulness. The present study hypothesizes that life satisfaction exerts an indirect positive path on mindfulness through these two mediators, even though its direct path becomes non-significant when they are included. The mediation model underscores the pivotal role of these two mediators in elucidating the relationship between life satisfaction and mindfulness.

## 4. Discussion

The present article hypothesizes that problematic online gaming and maladaptive daydreaming mediate the role of life satisfaction on mindfulness. Statistically significant relationships were also found between life satisfaction, problematic online gaming, maladaptive daydreaming, and mindfulness. These results are consistent with those of previous studies (Bajaj & Pande, 2016; Bargeron & Hormes, 2017; Mar et al., 2012; Müller et al., 2023; Phan et al., 2020; Sharma & Mahapatra, 2021).

This correlation analysis shows a highly significant correlation between online gaming and maladaptive daydreaming. This result is consistent with the literature (Öğüt, 2024; Sharma & Mahapatra, 2021; Zaheer et al., 2025). There may be several reasons for this similarity to the research literature; initially, online games are activities in which creativity is utilized to the most advanced capacity (Rahman et al., 2025). Furthermore, role-playing games are particularly common in esports (Bopp & Karadakis, 2023). Given that dreaming is important for role-playing, the correlation may have emerged as positive and significant. This is further supported by Bulut (2020), who suggests that daydreaming can lead to escapism. When examining problematic online gaming, the ‘escape’ sub-dimension is present (Pontes & Griffiths, 2015). In this context, it is worth noting that some sub-dimensions are similar, and this may act as a factor that weakens the correlation when measured as a total sub-dimension. Another important finding of the study is that although there is a positive correlation between life satisfaction and mindfulness, there is no direct path. This may be due to multiple causes. Mindfulness relates to concepts such as non-judgment, patience, clarity, and acceptance (Kabat-Zinn, 2003). While some subtopics exist in this context, they may not fully capture life satisfaction. Furthermore, considering the fact that life satisfaction varies even across age groups, this alone may not be sufficient to explain this.

The main hypothesis of the study is that problematic online gaming and maladaptive daydreaming mediate the role of life satisfaction on mindfulness. Despite the direct path being non-significant, when including problematic online gaming and maladaptive daydreaming in the model, it produces a statistically significant positive result. Testing the model revealed that all the path-analysis paths (a^1^b^1^a^2^b^2^) were significant. Although this is the first parallel mediation research study, the model’s path analyses are consistent with previous studies (Müller et al., 2023; Makas & Koç, 2025; Mar et al., 2012; Sharma & Mahapatra, 2021; Öğüt, 2024). There may be multiple reasons for similar results, but to give an example, it offers important findings that will increase the awareness of players interested in esports. To illustrate the relevant model, online esports-involved gamers who realize that they are dissatisfied with their school life, family, and friends and consequently turn to online games may experience a decrease in life satisfaction and an increase in both problematic online gaming and gaming time. When not gaming, this can lead to maladaptive daydreaming and decreased mindfulness when alone. Consequently, an individual’s inability to stay present while gaming or to be mindful of their emotions and thoughts can lead to a decrease in mindfulness. This opinion is similar to this study’s results.

Additionally, as mental health problems such as maladaptive daydreaming and problematic online gaming become more common among esports-involved gamers (Sharma & Mahapatra, 2021; Öğüt, 2024; ELNahas et al., 2018; Wang et al., 2025), their conscious awareness will decrease significantly, and the reasons for the decline in their mindfulness will become difficult to understand. Therefore, increasing conscious mindfulness by staying in the moment and maintaining this mindfulness throughout life can contribute to individuals becoming healthier, both psychologically and physiologically. For this reason, it can be stated that the study to be done, especially on esports-involved gamers, is important.

## 5. Limitations

This research has several limitations. First, it is a cross-sectional study, and the principle of such studies is that they cannot identify causality (Maxwell & Cole, 2007). For this reason, terms such as “causality” are not included in the study. To sum it up, a limitation is that cross-sectional mediation analyses and the definitive demonstration of causal relationships are limited. A further limitation is that some of the variables (life satisfaction, mindfulness, problematic online gaming and maladaptive daydreaming) are treated as dependent variables, whilst others are treated as independent variables. In this study, life satisfaction is treated as an independent variable; however, it is also suggested that it be examined as a dependent variable. Third, the sample is limited to individuals who play online games, and the study does not include information about the duration or experiences of gaming. Another limitation is the use of self-report measurement. Finally, the fact that it was carried out in Türkiye is the biggest limitation.

## 6. Practical Implications

This research makes a significant contribution by presenting a comprehensive model that integrates mediating models to describe problematic mindfulness. There are several reasons why dealing with life satisfaction as an independent variable in the model is important in terms of practical implications. Firstly, a decrease in life satisfaction may occur due to factors other than problematic online gaming, stemming from a variety of technology-based behaviors. For example, online gambling (Koivula et al., 2022; Huțul et al., 2024), smartphone addiction, and the fear of missing out (FOMO) (Tufan et al., 2026) are some of these. According to a longitudinal study, a lack of physical activity during leisure time can even decrease life satisfaction (Fan & Lim, 2026). It is important to test the direct effect of increasing such a complex structure on maladaptive daydreaming and problematic online gaming, and consequently to increasing mindfulness. Furthermore, the problem of playing games as a mediator and the technology-based aspects of game development may also be of significance to game developers. For instances, redefining age classifications in esports games in particular could serve as a guide when taking behavioral feedback into account. It could also help to address issues that may arise from long-term use.

However, from a clinical perspective, it should be noted that the implications may be limited. It should be noted that the validity and reliability of the relevant scales have also been tested in non-clinical samples in Turkey. This is mentioned in the section on data collection tools in the study. Given that mental health professionals have been shown to reduce problematic online gaming and enhance mindfulness skills through mindfulness-based interventions (Bekir, 2025), it is important to prevent such issues before a negative process develops. By addressing groups at potential risk, life satisfaction can be maintained at a high level, and opportunities can be provided to intervene before negative (maladaptive daydreaming and problematic gaming) behaviors develop. However, it is important to exercise caution due to the risk that different results may emerge in a clinical sample.

## 7. Conclusions

The total indirect roles of the parallel multiple mediator model indicated that for esports-involved gamers, problematic online gaming and maladaptive daydreaming mediated the relationship between life satisfaction and mindfulness. As a result, it is important to study the variables in the study together. Therefore, mental health professionals should identify levels of problematic online gaming and maladaptive daydreaming in esports-involved gamers before establishing strategies to enhance mindfulness and promote psychological well-being.

## Figures and Tables

**Table 1:** Descriptive statistics, linearity, normality and multicollinearity and correlations.

Variables	N	Min.	Max.	Mean	SD	Skew.	Kurt.	VIF	CI	1	2	3	4
1. Mindfulness	480	16	83	56.99	13.78	−0.69	0.33			1			
2. Problematic online gaming	480	9	41	22.22	6.32	0.36	−0.12	1.34	5.89	−0.24 **	1		
3. Maladaptive daydreaming	480	16	60	28.14	7.56	0.92	0.22	1.36	10.38	−0.23 **	0.50 **	1	
4. Life satisfaction	480	5	25	15.40	4.62	0.01	−0.69	1.05	13.99	0.12 **	−0.16 **	−0.20 **	1

** = *p* < 0.05.

**Table 2:** Parallel mediational model coefficients.

	M1				M2				Y (Mindfulness)			
**Predictors**		β	B	*p*		β	B	*p*		β	B	*p*
Life Satisfaction (X)	a_1_	−0.20	−34.	0.00	a_2_	−0.16	−0.22	0.00	c′	—	0.19	0.14
**Maladaptive Daydreaming (M1)**	—	—		—	—	—		—	b_1_	—	−0.23	0.01
Problematic Online Gaming (M2)	—	—		—	—	—		—	b_2_	—	−0.37	0.00
**Constant**	—		33.38	0.00	—		25.73	0.00	—		68.83	0.00
	R^2^ = 0.04				R^2^ = 0.02				R^2^ = 0.08			
	F (1, 478) = 21.58, *p* < 0.00				F (2, 478) = 13.59, *p* < 0.00				F (3, 476) = 13.78, *p* < 0.00			

**Table 3:** Bootstrapping process of the partial model.

Pathways						
**Direct Effects**	Effect	SE	*t*	*p*	BootLLCI	BootULCI
Life Satisfaction → Maladaptive Daydreaming	−0.34	0.07	−4.64	0.00	−0.48	−0.19
Life Satisfaction → Problematic Online Gaming	−0.22	0.06	−3.68	0.00	−0.34	−0.10
Life Satisfaction → Mindfulness (c′)	0.19	0.13	1.46	0.14	−0.06	0.46
Maladaptive Daydreaming → Mindfulness	−0.23	0.09	−2.53	0.01	−0.42	−0.05
Problematic Online Gaming → Mindfulness	−0.37	0.11	−3.33	0.00	−0.58	−0.15
**Total and Indirect Effects**	Effect	SE/BOOT			LL	UL
Total Effect (X **→** Y)	0.36	0.13			0.09	0.62
Total Indirect Effect	0.16	0.04			0.08	0.26
Indirect via Maladaptive Daydreaming	0.08	0.03			0.01	0.16
Indirect via Problematic Online Gaming	0.08	0.03			0.02	0.17

## Data Availability

Data is available upon reasonable request.
